# Supplementary material for: Cleavage of the Perlecan-Semaphorin 3A-Plexin A1-Neuropilin-1 (PSPN) Complex by Matrix Metalloproteinase 7/Matrilysin Triggers Prostate Cancer Cell Dyscohesion and Migration
Source: Int J Mol Sci. 2021 Mar 22;22(6):3218. doi: 10.3390/ijms22063218 (PMC8004947; doi:10.3390/ijms22063218)
Supplement: Supplementary file 1 [file ijms-22-03218-s001.pdf]

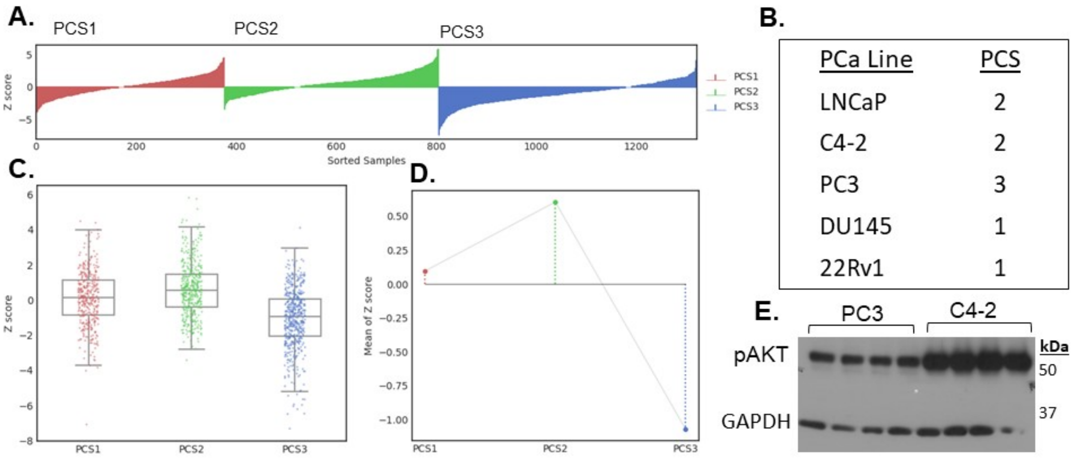

Supplemental Figure 1. Analysis of PI3K-AKT pathway by PCa subtype in the Prostate Cancer Transcriptome Atlas (PCTA) (http://www.thepcta.org/). An integrated analysis of gene expression in prostate cancer tissues and 8 human PC cell lines from The Cancer Cell Line Encyclopedia (CCLE) allows pathway analysis by query. As shown in the images extracted from this database, the PI3K-AKT pathway that we found to be activated by MMP-7 is most active in cell lines that are PCS2, including C4-2, a castrate resistant derivative line from LNCaP, chosen for mechanistic study in this work. Panels A, C and D represent lollipop, box, and lineplot of mean trend values of the PI3K-AKT pathway from the database. The Z score is a mathematically calculated measure of the difference between the error-weighted mean of the expression values of the genes in a gene signature and the error-weighted mean of all genes in a sample after normalization. Panel E shows by Western blot the relative levels of active pAKT that we measured in PC3 (PCS3) and C4-2 (PCS2) cells, consistent with the database inquiry. Other commonly used cell lines (panel B) represent different subtypes from the LNCaP series and are less well suited to analysis of the proteolytic regulation of the PSPN pathway. PCS subtypes were analyzed in reference 9.

Supplementary Table 1 (S1). MMP-7 levels and prostate cancer subtype. MMP-7 levels (ng/mL), prostate cancer subtype (PCS) and clustering behavior.

Samples	MMP-7 Levels in Conditioned Media	Prostate Cancer Subtype	Dm IV-3 Response
FBS media	Not Detectable	N/A	N/A
LNCaP	0.07 ng/mL	PCS2	Clusters
LNCaP RANKL	2.23 ng/mL	unknown	No Clusters
C4-2	Not Detectable	PCS2	Clusters
PC-3	0.21 ng/mL	PCS3	Small Clusters

Supplementary Table 2 (S2): Repeated Measures two-way ANOVA results (GraphPad Prism 9.0.1)

ANOVA table	SS	DF	MS	F (DFn, DFd)	P value
Time x Treatment	49	15	3.27	F (15, 135) = 4.949	P<0.0001
Time	561	3	187	F (1.345, 60.53) = 283.4	P<0.0001
Treatment	237	5	47.4	F (5, 45) = 6.268	P=0.0002
Subject	340	45	7.55	F (45, 135) = 11.44	P<0.0001
Residual	89.2	135	0.66		

P values indicate the following:
The population means of Time are significantly different.
The population means of Treatment are significantly different.
The interaction between Time and Treatment is significantly different.
